# Effects of iron deficiency on transfusion requirements in critically ill patients: a preliminary observational study

**DOI:** 10.1186/cc14419

**Published:** 2015-03-16

**Authors:** M Aydogan, M Ucar, A Yücel, B Karakas, A Gok, T Togal

**Affiliations:** 1 Inonu University, Malatya, Turkey

## Introduction

Critically ill patients often need blood transfusion, but no reliable predictors of transfusion requirements are available at ICU admission. We hypothesized that ICU patients admitted with iron deficiency may be at higher risk for developing anemia, requiring blood transfusion. The aims of this study were to determine the frequency of iron deficiency in ICU patients at admission and to investigate its relationship with transfusion requirements in ICU patients.

## Methods

Eighty-five patients admitted to the general ICU were enrolled in the prospective observational study. We studied 58 patients, after excluding those transfused on or before ICU admission. The patients' age, gender, APACHE II score, diagnosis, severity score, presence of sepsis, ICU complications, ICU treatments, and transfusion-free interval were recorded. Iron deficiency was assessed on the basis of several parameters, including hemoglobin, hematocrit, levels of serum iron, iron-binding capacity, transferrin saturation, levels of ferritin, soluble transferrin receptor, hepcidin, C-reactive protein, and peripheral blood smear.

## Results

The mean age was 43.5 ± 5.7 years. Of 58 patients (38 male/20 female), 25 (43.1%) had iron deficiency with outcomes of blood samples used at ICU admission. The overall transfusion rate was 32.8%, being higher in iron-deficiency patients than in normal iron profile patients (42.3 vs. 14.9%, *P *= 0.001). After adjusting for severity of illness and hemoglobin level, iron-deficiency patients remained significantly associated with transfusion, with a hazard ratio of 4.2 (95% CI, 1.3 to 12.9; *P *= 0.001).

## Conclusion

Iron deficiency is common at ICU admission and is associated with higher transfusion requirements. These findings have important implications for transfusion practices in ICU patients.

